# Biosynthesis and Antibacterial Activity of Silver Nanoparticles Using Yeast Extract as Reducing and Capping Agents

**DOI:** 10.1186/s11671-019-3244-z

**Published:** 2020-01-16

**Authors:** Mengjun Shu, Fengjiao He, Zhaohui Li, Xingzhong Zhu, Yujie Ma, Zhihua Zhou, Zhi Yang, Feng Gao, Min Zeng

**Affiliations:** Key Laboratory of Thin Film and Microfabrication (Ministry of Education), Department of Micro/Nano Electronics, School of Electronic Information and Electrical Engineering, Shanghai Jiao Tong University, Shanghai, 200240 People’s Republic of China

**Keywords:** Silver nanoparticles, Biosynthesis, Yeast Extract, Stability, Antibacterial activity

## Abstract

Biosynthesis for the preparation of antimicrobial silver nanoparticles (Ag NPs) is a green method without the use of cytotoxic reducing and surfactant agents. Herein, shape-controlled and well-dispersed Ag NPs were biosynthesized using yeast extract as reducing and capping agents. The synthesized Ag NPs exhibited a uniform spherical shape and fine size, with an average size of 13.8 nm. The biomolecules of reductive amino acids, alpha-linolenic acid, and carbohydrates in yeast extract have a significant role in the formation of Ag NPs, which was proved by the Fourier transform infrared spectroscopy analysis. In addition, amino acids on the surface of Ag NPs carry net negative charges which maximize the electrostatic repulsion interactions in alkaline solution, providing favorable stability for more than a year without precipitation. The Ag NPs in combination treatment with ampicillin reversed the resistance in ampicillin-resistant *E. coli* cells. These monodispersed Ag NPs could be a promising alternative for the disinfection of multidrug-resistant bacterial strains, and they showed negligible cytotoxicity and good biocompatibility toward Cos-7 cells.

## Introduction

Drug-resistant infections are a major cause of death and have resulted in a serious risk to public health. Additionally, increasing resistance to antimicrobial drugs is emerging as an urgent problem in medicine [1]. A number of strains of *Staphylococcus aureus* are resistant to methicillin and are the major cause of acquired infections in hospitals. Furthermore, other antibiotic-resistant bacteria include penicillin-resistant *Neisseria gonorrhoeae* and multidrug-resistant *Escherichia coli* (*E. coli*) [2, 3]. The major mechanisms of resistance are increased efflux and the reduced absorption of antibiotics [4]. Another mechanism of drug resistance is the expression of enzymes that modify the molecular structure of antibiotics [5]. Although much effort has been focused on developing the next generation of antimicrobial agents, there is an increased need for superior disinfection methods.

Silver nanoparticles (Ag NPs) have been used in many applications, such as protein carriers, radiosensitizers, solar fuel cell efficiency improvement, and antibacterial agents [6–8]. Nanoparticles, including metal-containing nanoparticles, Ag NPs are the most widely used as antimicrobial agents [9]. In reality, silver nanoparticles have shown significant antimicrobial activity against bacterial strains but negligible cytotoxicity to animal cells [10, 11]. Moreover, Ag NPs have exhibited antimicrobial activity against fungi, certain viruses, and antibiotic-resistant bacterial strains. With regard to their mechanism of action, suppression of DNA replication, blockage of the electrical potential difference needed in cytoplasmic membranes, and suppression of the respiratory chain are the main mechanisms of action of Ag NPs. Thus, the size, surface structure, and controlled shapes of Ag NPs play crucial roles in their antimicrobial activity and other applications. The general method for the preparation of Ag NPs involves the reduction of silver ions in the presence of an appropriate surfactant to achieve the controlled growth of Ag NPs [12]. The majority of reducing and surfactant agents show cytotoxicity to human tissue cells and potentially cause environmental contamination. Therefore, more effort in developing green methods for the preparation of shape-controlled Ag NPs is essential.

In this work, we present a novel route for the biosynthesis of Ag NPs by utilizing yeast extract. During the process, yeast extract supplies reducing and capping agents, including amino acids, vitamins, and carbohydrates, whereas silver ions serve as an electron acceptor. As a result, the favorable stability provided by the organic capping agents on the surface, the monodispersed Ag NPs, can be preserved for more than a year without precipitation. It was found that Ag NPs displayed a superior antibacterial activity compared to ampicillin against ampicillin-resistant *E. coli* cells. Compared to conventional synthetic methods, the biosynthesis approach presented herein is biocompatible, cost-effective, and environmentally benign. Furthermore, the shape-controlled and well-dispersed Ag NPs displayed good antibacterial effects toward *E. coli*.

## Methods

### Materials

Silver nitrate (AgNO_3_), sucrose (C_12_H_22_O_11_), sodium chloride (NaCl), and sodium hydroxide (NaOH) were purchased from Sinopharm Co., Ltd. Dry baker’s yeast was obtained from AB/MAURI Co., Ltd. *E. coli* was purchased from TransGen Biotech Co., Ltd. The CellTiter 96® Aqueous One Solution Cell Proliferation Assay kit (MTS) was purchased from Promega Biotech Co., Ltd. pcDNA3.4 plasmid, 1 × NuPAGE® LDS sample buffer, Dulbecco’s modified Eagle medium (DMEM) and fetal bovine serum (FBS) were purchased from Thermo Fisher Scientific Inc. Ampicillin and Luria-Bertani (LB) medium were purchased from Sangon Biotech Co., Ltd. All chemicals were analytical reagents and used without further purification. Deionized ultrapure water (18.2 MΩ.cm) was used throughout the experiments.

#### Synthesis of Ag NPs

The stocked yeast cells were inoculated into Luria-Bertani (LB) medium and shaken at approximately 150 rpm overnight at 25 °C for activation. Then, the activated yeast cells were washed with 0.9% saline solution and dispersed in 2% sucrose solution with shaking at approximately 150 rpm for 6 h at 25 °C. Finally, the cell-free yeast extract was collected for the biosynthesis of Ag NPs by centrifugation at 2000 rpm for 5 min. During the biosynthetic process, the pH value of yeast extract was adjusted to 10 with a NaOH solution, and then, the AgNO_3_ solution was gradually added to the above solution under vigorously magnetic stirring. At last, the obtained Ag NPs were dialyzed with 1 kDa dialysis membranes for 5 days and freeze-dried for further characterization.

#### Characterizations

Transmission electron microscopy (TEM) images of Ag NPs were observed on JEM-2100 microscope with an accelerating voltage of 200 kV (JEOL, Japan). Scanning electron microscopy (SEM) images were obtained on a Carl Zeiss ULTRA plus scanning electron microscope (Carl Zeiss, Germany) equipped with an energy dispersive spectrometer (EDS) operated at 20 kV. Ultraviolet-visible (UV-Vis) absorption spectra were recorded on a Lambda 950 UV/Vis/NIR spectrophotometer (Perkin-Elmer, USA). X-ray powder diffraction (XRD) patterns were obtained using a D8 Advance instrument (Bruker, Germany). Fourier transform infrared spectroscopy (FTIR) was recorded from 4000-500 cm^−1^ with samples prepared as KBr pellets on a Vertex 70 FTIR spectrometer (Bruker, Germany). The zeta-potential of Ag NPs was measured with a Malvern Zeta Nano ZS-90 (Malvern, United Kingdom) at 25 °C. The surface elements on Ag NPs were identified by X-ray photoelectron spectroscopy (XPS) using a Kratos AXIS Ultra DLD instrument with a monochromatic Al Kα source (1486.6 eV) (Shimadzu, Japan). The amino acid components were analyzed with an L-8900 high-speed amino acid analyzer (Hitachi, Japan).

#### Cell Cytotoxicity Assay

To explore the biocompatibility of the prepared Ag NPs, an MTS assay was employed to evaluate the cell cytotoxicity of the Ag NPs [13]. Cos-7 cells were cultured in DMEM supplemented with 10% FBS complete medium in a humidified atmosphere incubator containing 5% CO_2_ at 37 °C. The cells were plated into 96-well flat-bottomed plates at a density of 10000 cells per well and cultured for 24 h. Then, the growth medium was replaced with fresh DMEM medium containing different concentrations of Ag NPs. After incubation for another 24 h, the relatively viable cells were determined by MTS. The absorbance was measured at 490 nm using a SpectraMax® M5 microplate reader (Molecular Devices, USA). Nontreated cells in DMEM medium were used as a control.

#### SDS-PAGE Assay

Standard SDS-PAGE was performed with a 10% (w/v) separating gel and a 4% stacking gel. The samples were boiled for 5 min with 1 × NuPAGE® LDS Sample Buffer and centrifuged at 12000 rpm for 5 min before application to the gels. The standard protein marker was used as a reference control. The gels were stained with 0.5% Coomassie Blue. Images of gels were recorded with GelDoc XR^+^ gel imaging systems (Bio-Rad, USA).

#### Antimicrobial Activity Studies

To determine the antimicrobial activity, the synthesized Ag NPs were tested for bactericidal activity against *E. coli* [14]. A single colony of *E. coli* was grown overnight at 37 °C in LB medium on an orbital shaker at 150 rpm. Colonies were adjusted to an OD of 0.01–0.02 at 600 nm with fresh LB medium. Then, 100 μL of serial dilutions of Ag NPs were filled onto 96-well microplates. The microplates were then inoculated with 100 μL of diluted *E. coli* solution and incubated for 16 h at 37 °C. The viability of *E. coli* was determined by the measurement of the absorbance at 600 nm with a SpectraMax® M5 microplate reader (Molecular Devices, USA). A time-course analysis was performed to evaluate the antibacterial sensitivity against *E. coli* over time. Finally, 100 μL of *E. coli* solution was added to sterile tubes containing 10 and 20 μg/mL Ag NPs, respectively. The absorbance at 600 nm was measured with a SpectraMax® M5 microplate reader (Molecular Devices, USA) after 1, 2, 4, and 6 h.

Colony-forming unit assay was introduced to investigate the Ag NPs against the antibiotic-resistant bacterial cells. *E. coli* stably expresses pcDNA3.4 plasmid containing the β-lactamase gene which confers resistance to ampicillin as a model. When the ampicillin-resistant *E. coli* (*E. coli*-Amp^+^) cells reached log phase growth, the *E. coli*-Amp^+^ cells were grown in the LB agar plate in the treatment with ampicillin alone or in the combinational treatment with Ag NPs and incubated at 37 °C for 18 h. The number of *E. coli*-Amp^+^ colonies formed on LB plates was calculated. All assays were performed at least three times.

## Results and Discussion

### Synthesis of Ag NPs

**Scheme 1:**
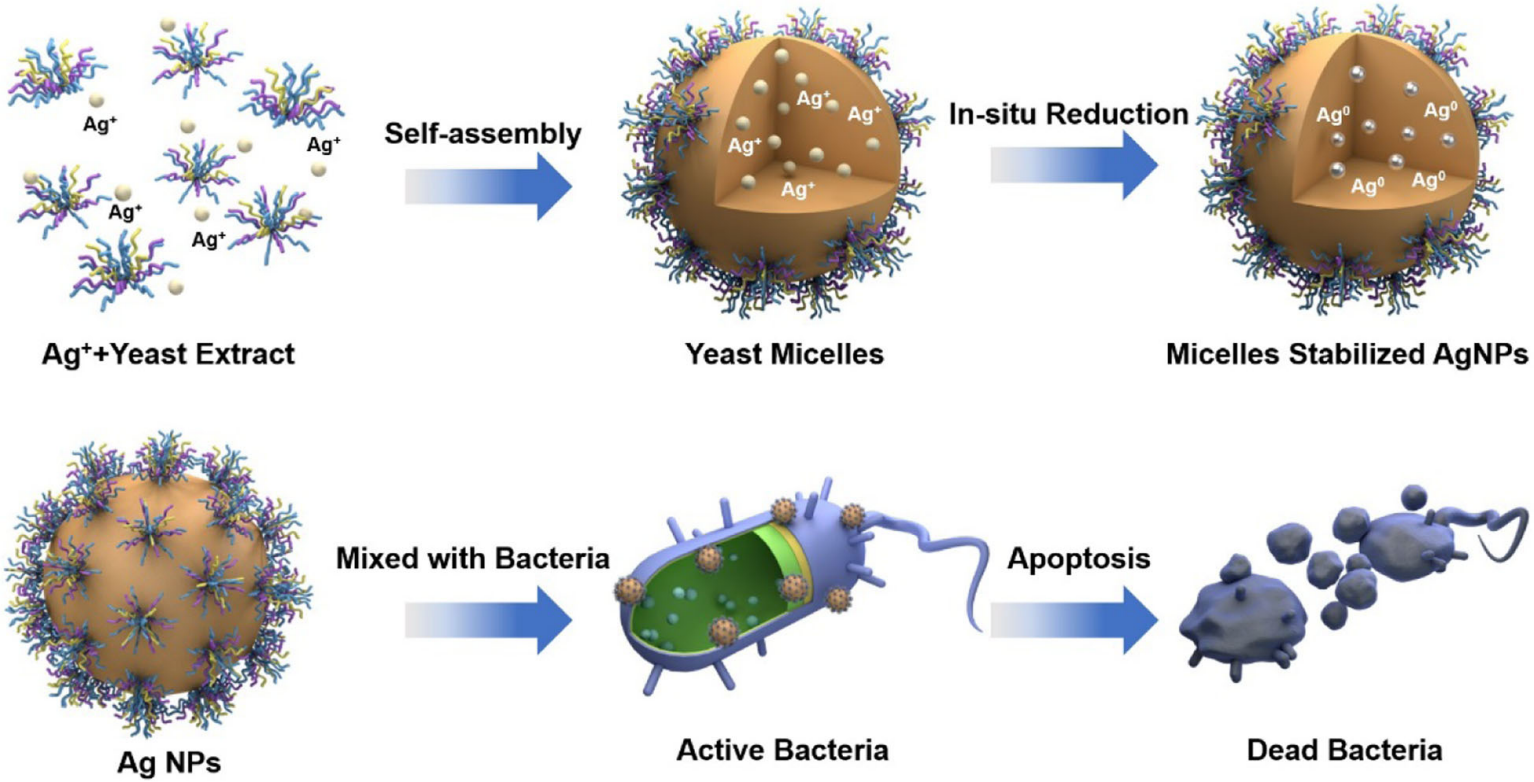
Proposed schematic illustration of the biosynthesis of Ag NPs

As schematically illustrated in Scheme 1, the preparation of Ag NPs started with the self-assembly of biomolecules in the yeast extract to form yeast micelles. Then, Ag^+^ was reduced in situ by the reducing agents in the yeast extract, including amino acids, vitamins, and carbohydrates. The formed Ag nanoparticles were stabilized by the biomolecules. The surface coating on Ag NPs enhanced the affinity towards the bacterial membrane, increasing the permeability of the cell wall. The interaction between Ag NPs and peptidoglycan changed the configuration of peptidoglycan, finally leading to the apoptosis process to damage the bacteria.

### Structural Characterization of Ag NPs

**Fig. 1:**
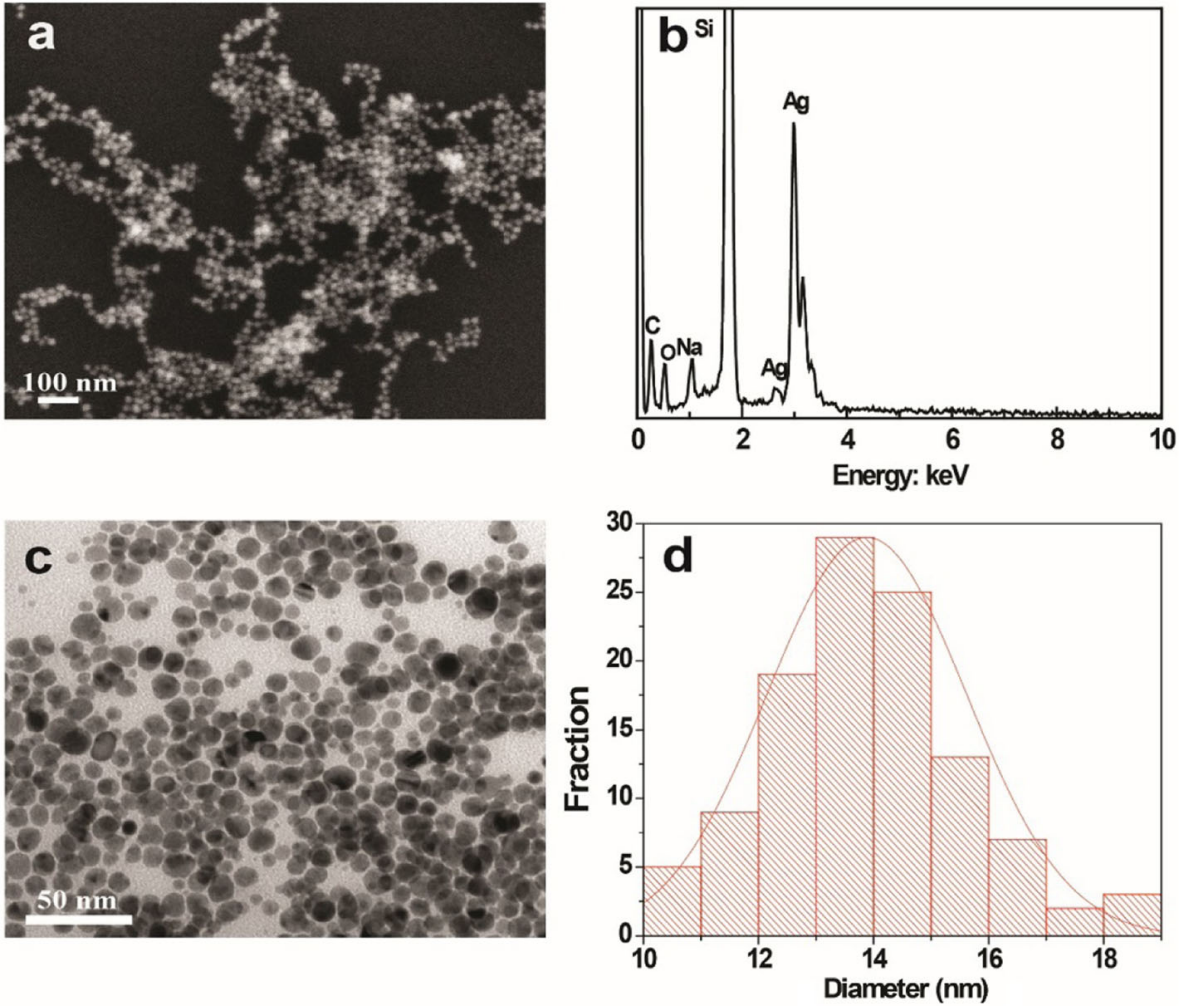
**a** Field emission SEM image of Ag NPs, **b** EDX spectrum of Ag NPs, **c** TEM image of Ag NPs, and **d** size distribution of Ag NPs

**Fig. 2:**
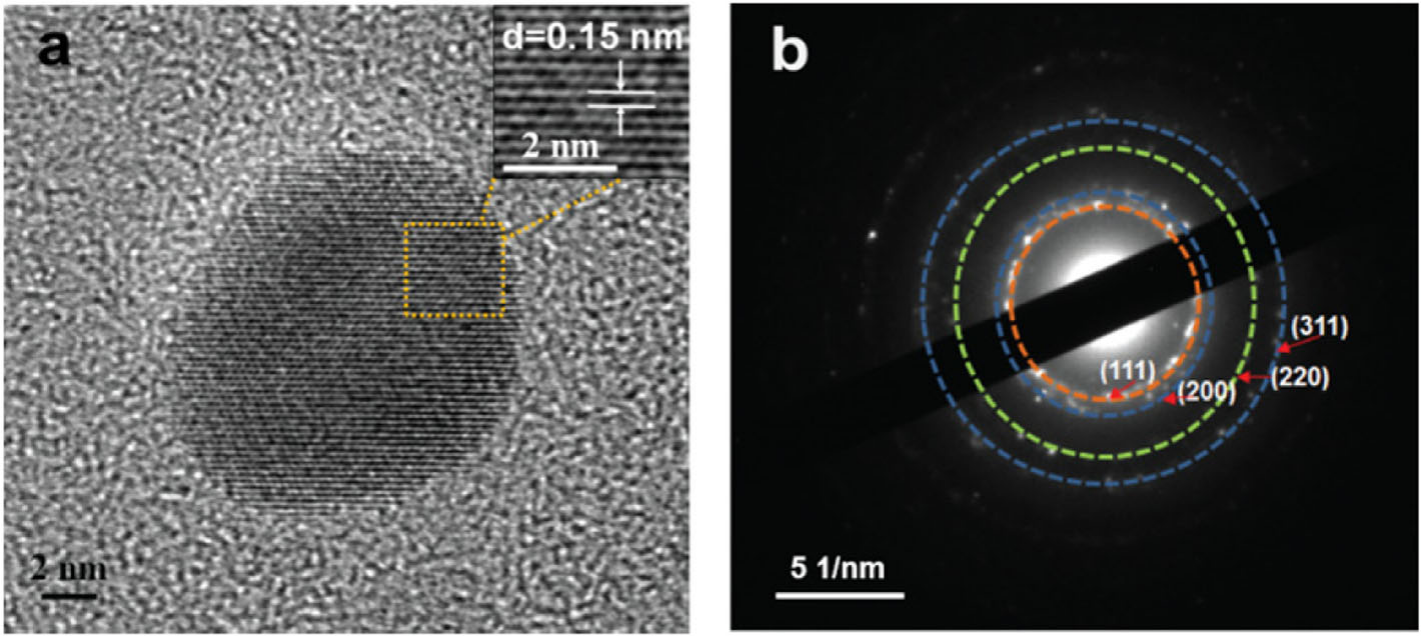
**a** Lattice fringes of Ag NPs in the HR-TEM image, **b** circular rings of Ag NPs from the typical selected-area diffraction (SAED) patterns

As shown in Fig. 1a, the typical SEM image showed that the synthesized Ag NPs have a spherical shape and fine size. The EDX confirmed the formation of Ag NPs (Fig. 1b). A strong optical absorption peak was observed at approximately 3 keV, which is a typical optical absorption peak of silver nanocrystallites for surface plasmon resonance. The minor amounts of oxygen and carbon could be attributed to the thin layer of organic capping on the synthesized Ag NPs. The reaction of AgNO_3_ solution with NaOH leads to the formation of a small amount of Ag_2_O. Therefore, a small amount of O can also be attributed to the presence of Ag_2_O. The morphology and size of the Ag NPs were further characterized by high-resolution TEM (HRTEM). The Ag NPs ranged in diameter from 10.3 to 18.9 nm (Fig. 1c), with an average size of 13.8 nm (Fig. 1d). The size, shape, and surface chemistry of Ag NPs showed an important effect on the antimicrobial activity. The smaller size and higher surface area allowed the Ag NPs to better interact with the bacterial membrane for further enhanced antimicrobial activity [15–17]. The clear lattice fringes in the HRTEM image showed a fringe spacing of 0.15 nm (Fig. 2a), which corresponds to the (220) planes of silver. As shown in Fig. 2b, the crystalline nature of the Ag NPs was demonstrated by the typical selected-area diffraction (SAED) patterns, where the bright circular rings correspond to the (311), (220), (200), and (111) planes [18, 19].

**Fig. 3:**
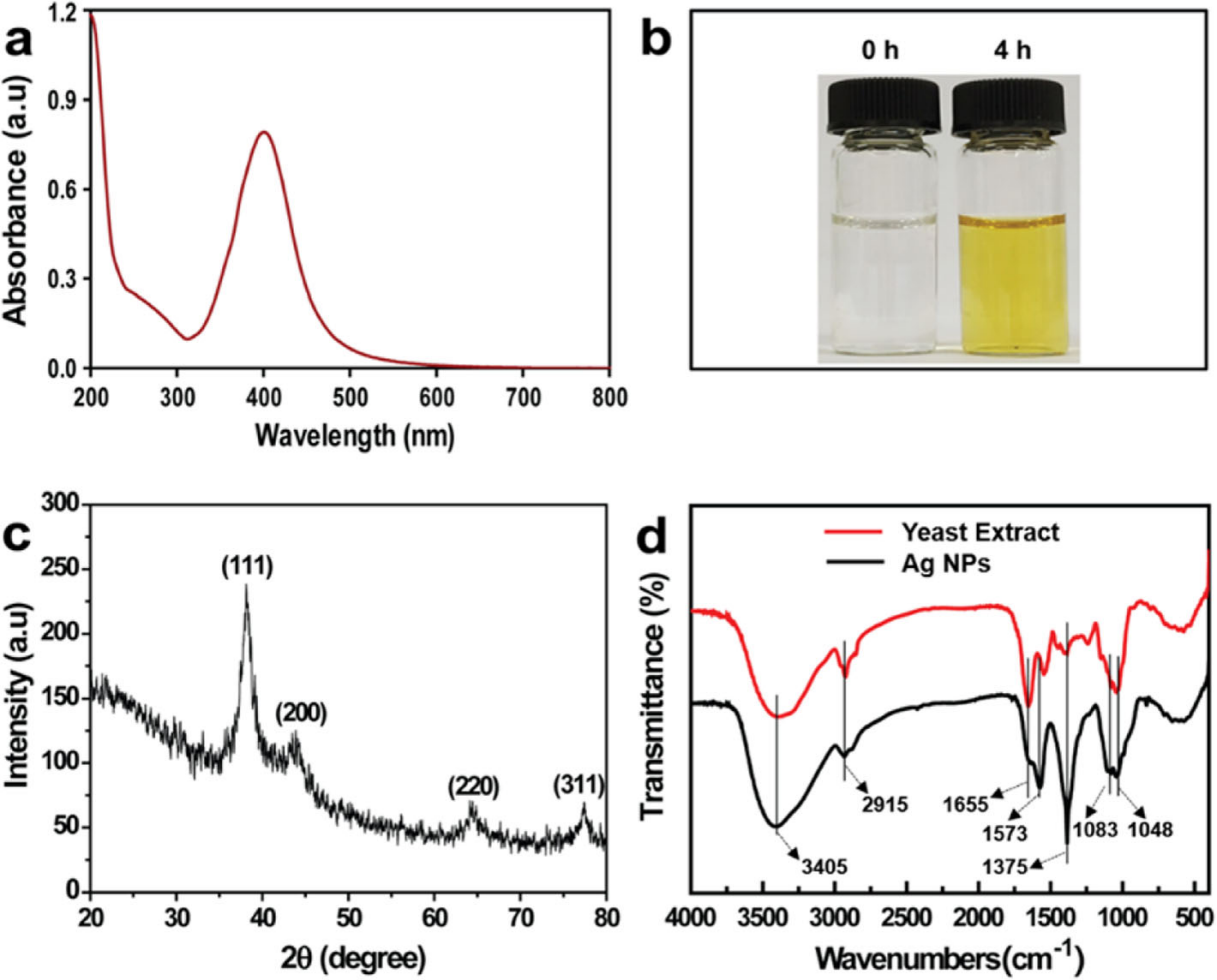
**a** UV-Vis spectrum of Ag NPs, **b** photo of synthesized Ag NPs, **c** XRD pattern of Ag NPs, and **d** FTIR spectrum of Ag NPs and yeast extract

**Fig. 4:**
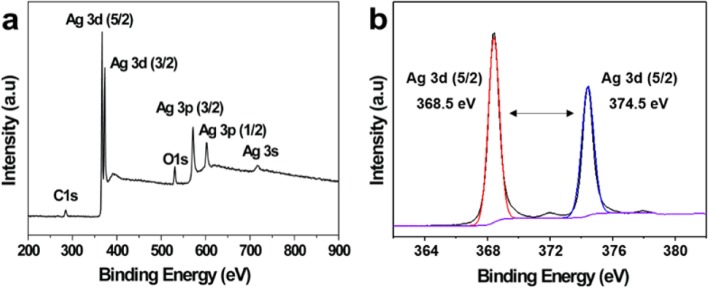
**a** The full scan of XPS spectrum of Ag NPs and **b** the Ag 3d XPS spectrum

The UV-Vis spectrum of Ag NPs exhibited a strong peak at 418 nm, which was due to surface plasmon resonance (Fig. 3a). A yellow solution of synthesized Ag NPs is shown in Fig. 3b, which indicates the formation of Ag NPs. The XRD pattern analysis of the synthesized Ag NPs showed four intense peaks at 77.36°, 64.30°, 43.52°, and 38.16°, corresponding to the (311), (220), (200), and (111) planes for silver, respectively (Fig. 3c). The data was confirmed by standard silver data from JCPDS card No. 04-0783 [20]. The XRD pattern demonstrated the crystalline nature of the synthesized Ag NPs, in agreement with a previous report [21]. FTIR analysis was employed to characterize and identify the potential biomolecules on the synthesized Ag NPs (Fig. 3d). The broad band at 3405 cm^−1^ corresponds to −OH stretching [22]. The weaker peak at 2915 cm^−1^ is assigned to the −CH2 stretching vibration. The band at 1655 cm^−1^ in the yeast extract is due to the C=O stretching vibration of carboxyl moieties, and this band shifts to 1573 cm^−1^ in Ag NPs, due to the interaction between carboxyl moieties and Ag NPs [23]. The sharp peak at 1375 cm^−1^ is attributed to the C–N stretching vibration. The bands at 1048 cm^−1^ and 1083 cm^−1^ are assigned to the stretching vibrations of C–O–C and C–OH, respectively [24, 25]. These results demonstrated that biomolecules of the yeast extract were responsible for the biosynthesis of Ag NPs. The surface coating on Ag NPs affected the affinity towards the bacterial membrane [26, 27]. The states of Ag NPs were further characterized by XPS. As shown in Fig. 4a, the full scan of the XPS spectrum with clear peaks was attributed to C 1*s*, Ag 3*d*, Ag 3*p*, Ag 3*s*, and O 1*s*. The Ag 3*d* (5/2) and Ag 3*d* (3/2) peaks were observed at binding energies of approximately 368.5 and 374.5 eV, respectively (Fig. 4b). This energy splitting value of 6.0 eV demonstrated the formation of Ag NPs [28, 29].

The surface charge of Ag NPs was determined by Malvern Zeta Nano ZS-90 instrument, which is an important parameter of stability and dispersion of the colloidal solutions. The zeta potential is the surface electrostatic potential at the boundary between the diffuse layer and compact layer of nanoparticles, and which is an indicator for applications of biomedical polymers [30]. As shown in Additional file 1: Fig. S1, at a lower pH value of 3, the zeta potential of Ag NPs revealed a slightly negative charge (− 3.2 mV). The zeta potential of Ag NPs decreased monotonically from − 12.1 mV at pH 7.0 to − 24.4 mV at pH 11.0, which confirmed the negatively charged groups on the surface of Ag NPs. Gao et al. reported that the dispersion and stability of Ag NPs mainly attribute to the surface charge [31]. The presence of negatively charged groups improves the stability and dispersion of Ag NPs in aqueous solutions [32].

### Cytotoxicity of Ag NPs and Analysis of Biomolecules

**Fig. 5:**
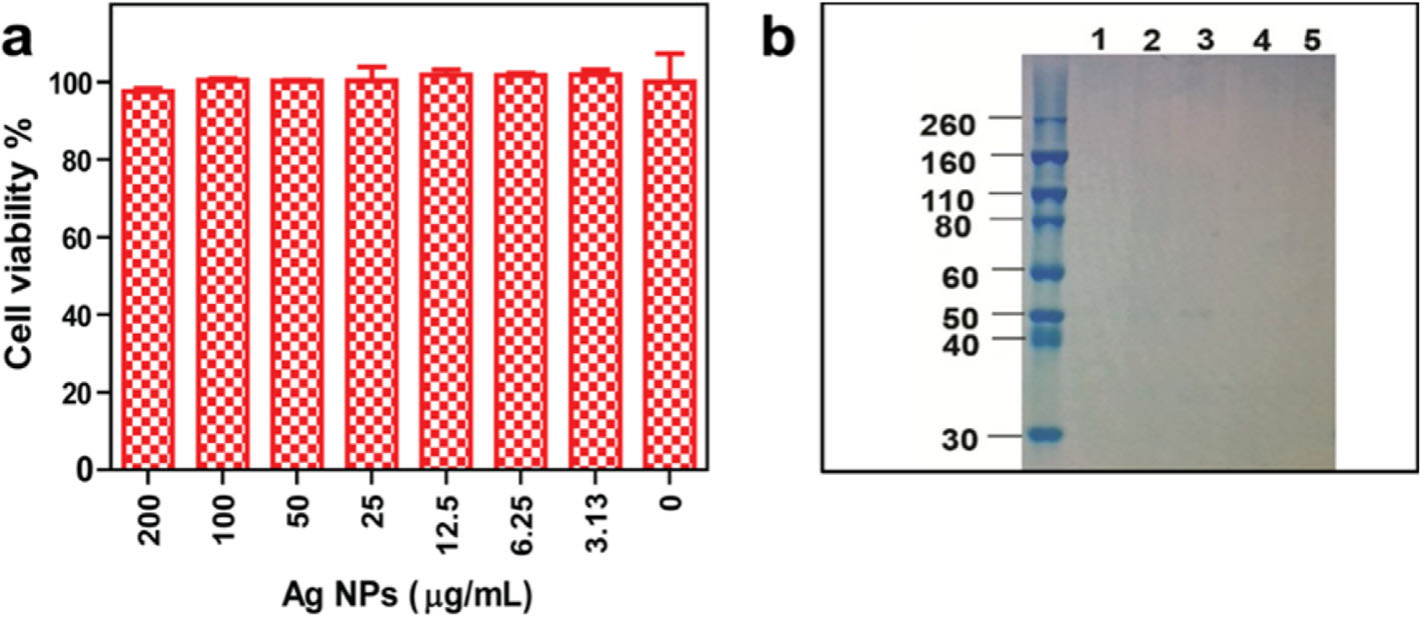
**a** Cytotoxicity of Ag NPs in Cos-7 cells and, **b** SDS-PAGE analysis. Lane 1: loading buffer control. Lanes 2–4: synthesized Ag NPs. Lane 5: yeast extract centrifuged with 8000 rpm

The biocompatibility of the synthesized Ag NPs is important for their further biomedical application. To investigate the cytotoxicity of the Ag NPs, the cell viability of Cos-7 cells was detected by the MTS assay. The Cos-7 cells were incubated with Ag NPs at different concentrations for 24 h. As shown in Fig. 5a, no significant cytotoxicity was revealed when cells were treated with the Ag NPs at concentrations as high as 200 μg/mL. It can be concluded that the Ag NPs showed negligible cytotoxicity and good biocompatibility towards Cos-7 cells.

To explore the synthetic mechanisms of the synthesized Ag NPs, we analyzed biomolecules on the surface of Ag NPs and yeast extract. As shown in Fig. 5b, the SDS-PAGE analysis showed no detectable or marginal protein on the surface of the synthesized Ag NPs or in the yeast extract. We further determined the biomolecules in the yeast extract with a high-speed amino acid analyzer. As summarized in Additional file 1: Table S1 of supporting information, there are approximately 22 kinds of amino acids in the yeast extract that are rich in glutamic acid, γ-aminobutyric acid, ornament, and alpha-linolenic acid. The isoelectric point of these amino acids is approximately 6, except those of lysine and arginine are approximately 10~11. In addition, a variety of components containing −NH_2_, such as urea, ammonia, asparagine, and glutamine, could be found. The biomolecules of reductive amino acids, alpha-linolenic acid, and carbohydrates in the yeast extract have a significant role in the formation of Ag NPs. It was reported that NADH-dependent reductase [33, 34] or the nitrate reductase enzyme is involved in the reduction process [35–37] in the biosynthesis of Ag NPs via the microorganism extract.

Biomolecules of the yeast extract play a decisive part in the formation of Ag NPs by protecting them from aggregation. Stabilizers of biomolecules help to prevent redundant reactions between Ag NPs [38]. The amphoteric molecules of amino acids contain both basic and acidic groups. The net charge of these amino acid compounds can be negative or positive depending on the pH changes of the yeast extract solution, which further affects the binding ability during the synthesis of Ag NPs [39]. In the alkaline solution, amino acids on the surface of Ag NPs carry net negative charges which maximize the electrostatic repulsion interactions [40–42]. The biomolecules from the yeast extract act as a capping agent and play a key role in controlling the size distribution, shape, and morphology in the formation of Ag NPs. The value of pH is an important factor with an effect on the controlled synthesis of Ag NPs in the study. When the pH value is below 7, nucleation occurs at a low rate. Ag NPs can be formed in a few minutes at higher pH values, and the particle size decreases with the increasing pH values of the solution. The optimal balance was demonstrated between the growth processes and nucleation [43]. The unstable and agglomerated Ag NPs always presented in the reduction process of solutions with extreme pH values (> 11) [44].

### Antibacterial Activity

**Fig. 6:**
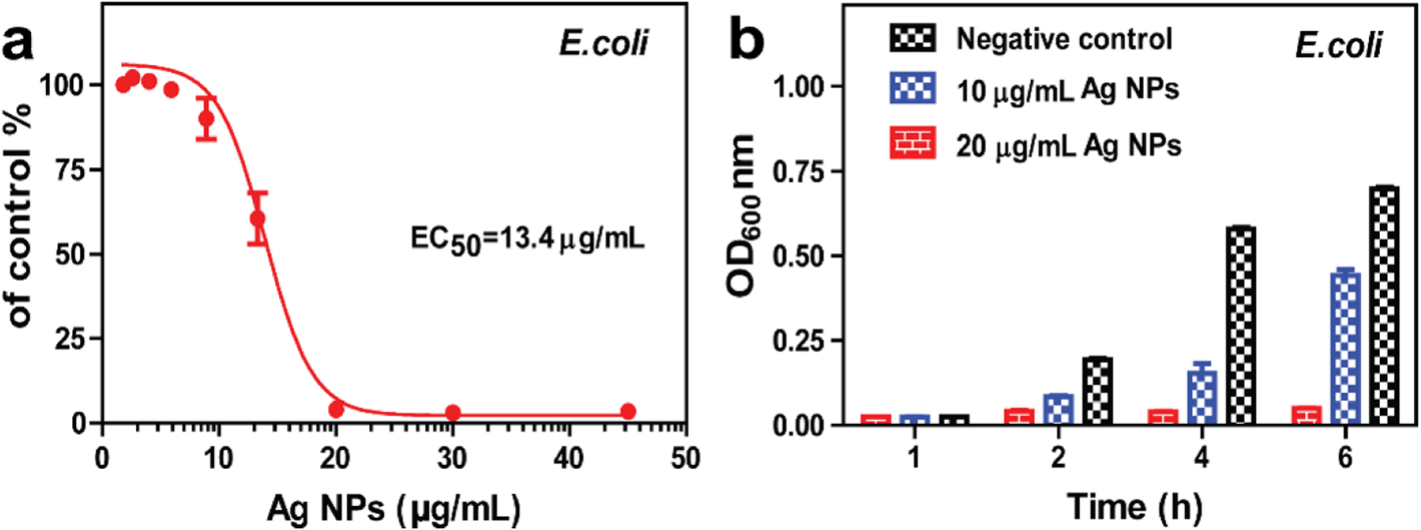
**a** The growth inhibition of *E. coli* and **b** time course analysis of the antibacterial effect

*E. coli* has been extensively evaluated for the antimicrobial activity of Ag NPs. The growth of *E. coli* in the presence or absence of Ag NPs proves the antimicrobial ability. As shown in Fig. 6a, the synthesized Ag NPs exhibited significant antibacterial activity in a concentration-dependent manner against *E. coli*. The growth inhibition assay demonstrated a complete reduction in *E. coli* at Ag NP concentrations above 20.0 μg/mL compared to the negative control. The half inhibitory concentration (EC_50_) of Ag NPs was 13.4 μg/mL. The dose of 20.0 μg/mL Ag NPs exhibited a significant antibacterial effect against *E. coli* throughout the tested time, while the 10.0 μg/mL Ag NPs showed a partial inhibitory effect (Fig. 6b).

**Fig. 7:**
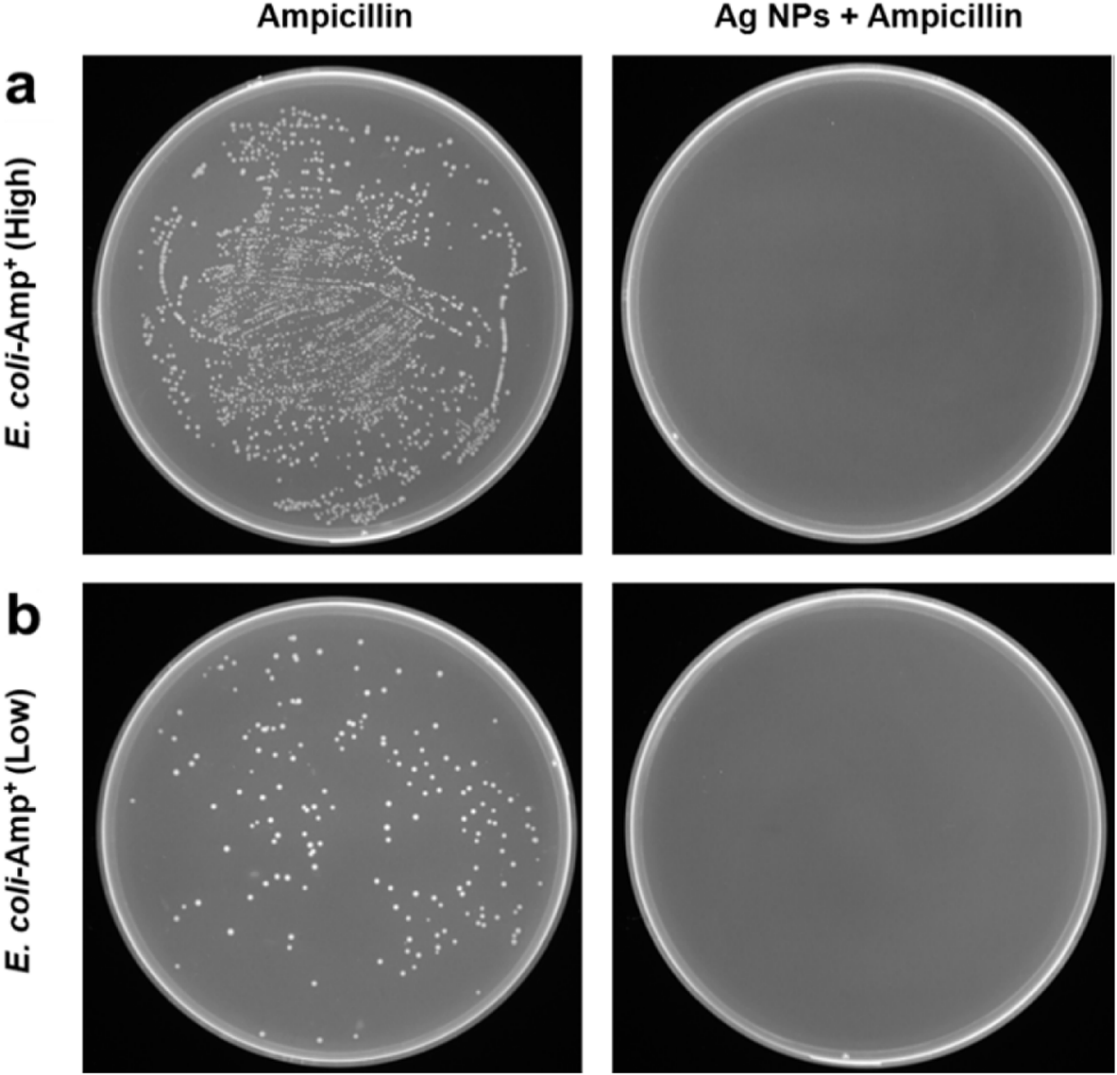
The growth of *E. coli*-Amp^+^ in treatment with ampicillin alone (50 μg/mL) or in combination with Ag NPs (25 μg/mL). **a** High density and **b** low density of *E. coli*-Amp^+^ cells

In order to investigate if the Ag NPs really affects the antibiotic-resistant bacterial cells, we evaluated the antibacterial activity of Ag NPs against ampicillin-resistant *E. coli* by colony-forming unit assay. *E. coli*-Amp^+^ stably expresses a high copy number of pcDNA3.4 plasmid containing the β-lactamase gene which confers ampicillin-resistance to *E. coli* [45]*.* The *E. coli*-Amp^+^ cells were grown in the LB agar plate in the treatment with ampicillin alone or in the combinational treatment with Ag NPs. The inhibitory activity of the prepared Ag NPs is presented in Fig. 7. It was noted that the Ag NPs in combination treatment with ampicillin displayed superior antibacterial activity compared to ampicillin alone. In contrast, the treatment of ampicillin alone has no inhibitory activity on *E. coli*-Amp^+^. Combination therapy of antibiotics and Ag NPs provides a complementary strategy to overcome antibiotic-resistant bacterial cells, which further improves the current therapeutic approaches. The overall results presented in this study contribute to the development of alternative antibacterial inhibitors to treat bacterial infections caused by multidrug-resistant bacterial strains.

There is a great need for novel drugs with different mechanisms to combat bacterial resistance. Due to their potent antimicrobial activity, Ag NPs have been used in medical products, personal care products and textiles. There are multiple mechanisms by which Ag NP combat microbial resistance [46]. Ag NPs accumulated on the bacterial membrane surface, increasing the permeability of the cell wall. The interaction between Ag NPs and peptidoglycan changed the configuration of peptidoglycan and thus damaged the bacterial membrane [47]. The characteristics of shape, surface structure, morphology, dispersity, and biocompatibility of Ag NPs have a significant role in their antimicrobial activity.

## Conclusions

Herein, we report a novel biosynthetic method for the preparation of Ag NPs using the yeast extract. The yeast micelles formed when the Ag^+^ solution was mixed with the yeast extract. Bioreducing biomolecules play a major role in the reduction of Ag^+^. In addition, the biomolecules provide favorable stability, monodispersity, and controllable size distribution for the synthesized Ag NPs, exhibiting good stability for more than a year without precipitation. The high-speed amino acid analysis revealed that the yeast extract is rich in biomolecules, including amino acids, alpha-linolenic acid, and aminobutyric acid. The Ag NPs exhibited significant antibacterial activity in a concentration-dependent manner against *E. coli*. The growth inhibition assay demonstrated a complete reduction in *E. coli* at concentrations of Ag NPs above 20.0 μg/mL. The Ag NPs in combination treatment with ampicillin exhibit superior antibacterial activity compared to ampicillin alone against ampicillin-resistant *E. coli* (*E. coli*-Amp^+^) cells. The surface coatings on Ag NPs enhanced the affinity towards the bacterial membrane and increased the permeability of the cell wall. The interaction between Ag NPs and peptidoglycan changed the configuration of peptidoglycan and finally led to the apoptosis of bacteria. Furthermore, these Ag NPs stabilized by the biomolecules exhibited low cytotoxicity and good biocompatibility toward Cos-7 cells.

## Supplementary information

**Additional file 1: Figure S1** Zeta potential of Ag NPs versus pH value. **Table S1** Summary of of biomolecules in yeast extract.

## Data Availability

The datasets supporting the conclusions of this current study are available from the corresponding authors upon reasonable request.

## Abbreviations

Ag NPs: Silver nanoparticles
DMEM: Dulbecco’s modified Eagle medium
*E. coli*: *Escherichia coli*
EDS: Energy dispersive spectrometer
FBS: Fetal bovine serum
FTIR: Fourier transform infrared spectroscopy
HRTEM: High-resolution TEM
LB: Luria-Bertani
SAED: Selected-area diffraction
SEM: Scanning electron microscopy
TEM: Transmission electron microscopy
UV-Vis: Ultraviolet-visible
XRD: X-ray powder diffraction
