# Supplementary material for: Biosynthesis and Antibacterial Activity of Silver Nanoparticles Using Yeast Extract as Reducing and Capping Agents
Source: Nanoscale Res Lett. 2020 Jan 16;15:14. doi: 10.1186/s11671-019-3244-z (PMC6965552; doi:10.1186/s11671-019-3244-z)
Supplement: Supplementary file 1 — Additional file 1: Figure S1 Zeta potential of Ag NPs versus pH value. Table S1 Summary of of biomolecules in yeast extract. [file 11671_2019_3244_MOESM1_ESM.docx]

Biosynthesis and Antibacterial Activity of Silver Nanoparticles Using Yeast Extract as Reducing and Capping Agents

Mengjun Shu^†^, Fengjiao He^†^, Zhaohui Li, Xingzhong Zhu, Yujie Ma, Zhihua Zhou, Zhi Yang*, Feng Gao* and Min Zeng*

Key Laboratory of Thin Film and Microfabrication (Ministry of Education), Department of Micro/Nano Electronics, School of Electronic Information and Electrical Engineering, Shanghai Jiao Tong University, Shanghai 200240, People’s Republic of China.

† The two authors contribute equally to this paper

**E-mail:** zhiyang@sjtu.edu.cn, fgao200906@126.com and minzeng@sjtu.edu.cn


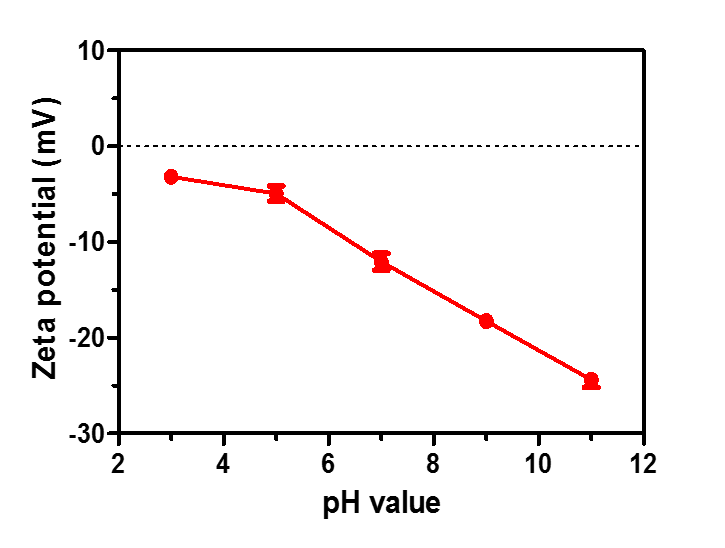


**Fig. S1** Zeta potential of Ag NPs versus pH value

**Table S1** Summary of biomolecules in Yeast Extract

| Name | (ng/mL) | Name | (ng/mL) |
| --- | --- | --- | --- |
| Urea | 2095.8 | Leucine | 2548.8 |
| Aspartic acid | 3844.8 | Tyrosine | 1507.7 |
| Threonine | 1889.7 | Phenylalanine | 1717.3 |
| Serine | 2988.3 | α-linolenic acid | 151.2 |
| Asparagine | 4269 | β-aminoisobutyric acid | 179.7 |
| Glutamic acid | 16273.2 | γ-aminobutyric acid | 7419.6 |
| Glutamine | 2871.8 | Ethanolamine | 91.3 |
| Glycine | 2049.5 | Ammonia | 955.7 |
| Alanine | 7032.2 | ornament | 1117.7 |
| Viline | 3026.2 | Lysine | 3471.4 |
| Cysteine | 223 | Histidine | 711.1 |
| Methionine | 578.3 | Arginine | 2657.6 |
| Isoleucine | 1898.4 |  |  |
